# Supplementary material for: Interactions between temperature and energy supply drive microbial communities in hydrothermal sediment
Source: Commun Biol. 2021 Aug 25;4:1006. doi: 10.1038/s42003-021-02507-1 (PMC8387401; doi:10.1038/s42003-021-02507-1)
Supplement: Supplementary file 2 — Supplementary Information [file 42003_2021_2507_MOESM2_ESM.pdf]

SUPPLEMENTARY MATERIALS

Interactions between temperature and energy supply drive microbial communities in hydrothermal sediment

Running title: Temperature and energy supply drive microbial communities

Lorenzo Lagostina[1], Søs Frandsen[2], Barbara J. MacGregor[3,4], Clemens Glombitza[1,2], Longhui Deng[1], Annika Fiskal[1], Jiaqi Li[1], Mechthild Doll[5], Sonja Geilert[6], Mark Schmidt[6], Florian Scholz[6], Stefano Michele Bernasconi[7], Bo Barker Jørgensen[2], Christian Hensen[6], Andreas Teske[3], and Mark Alexander Lever[1,2]*

[1]Institute of Biogeochemistry and Pollutant Dynamics, Eidgenössische Technische Hochschule Zürich, 8092 Zürich, Switzerland

[2]Center for Geomicrobiology, Department of BioScience, Aarhus University, DK-8000 Aarhus, Denmark

[3]Department of Marine Sciences, University of North Carolina at Chapel Hill, Chapel Hill, NC, 27599, USA

[4]Department of Earth and Environmental Sciences, University of Minnesota, Minneapolis, MN, 55455, USA

[5]Faculty of Geosciences (FB 05), University of Bremen, 28359 Bremen, Germany

[6]GEOMAR Helmholtz Centre for Ocean Research Kiel, Wischhofstraße 1-3, 24148 Kiel, Germany

[7]Department of Earth Sciences Eidgenössische Technische Hochschule Zürich, 8092, Zürich, Switzerland

*To whom correspondence should be addressed: Mark Alexander Lever, Eidgenössische Technische Hochschule Zürich, Institute of Biogeochemistry and Pollutant Dynamics, Universitätsstrasse 16, CHN G50.3, 8092 Zürich, Switzerland; phone: +41 44 632 85 27; email: mark.lever@usys.ethz.ch.

Supplementary Methods

Supplementary background on study area

The Guaymas basin is a young rift basin in the Gulf of California that is characterized by a highly productive water column, that supports high sedimentation rates of phytoplankton-derived organic matter to the seafloor, and vigorous seafloor and subseafloor hydrothermal activity driven by active seafloor spreading[27]. Tubular magmatic intrusions occur across the entire basin through the 200 to 400m thick organic-rich sediment column[15,27]. Magmatic intrusions into the organic-rich sediment lead the ‘thermogenic’ breakdown of sedimentary organic matter, which releases of methane, short chain organic acids (SCOAs), aliphatic and aromatic hydrocarbons, including petroleum compounds, and labile protein and carbohydrate-derived organic matter[1,2,16,26,30,38,42]. In addition, the high temperatures caused by magmatic intrusions result in sulfide production via the thermochemical reduction of sulfate at temperatures >110°C[19]. In certain regions, vertically advecting fluids transport these compounds to shallow sediments with temperatures in the growth range of microbial life, where they sustain vast populations of chemoorgano- and chemolithoautotrophic microorganisms, including dense mats of sulfur-oxidizing bacteria (*Beggiatoaceae*)[31,35,41]. These microorganisms in turn form the basis of a rich food web that supports a high biomass of macrofauna[20,39]. Vertical fluid advection also leads to formation of hard structures at the seafloor, including hydrothermal vent chimneys and carbonate crusts[12,47].

The abundant supplies of diverse energy substrates combined with high diversity of sedimentary habitats[47], and the extreme spatial and temporal variability in temperature[31,32] results in a dynamic sedimentary environment that supports functionally, physiologically, and phylogenetically very diverse microbial communities[14,24,44]. Decades of microbiological cultivation have led to the isolation of many thermophilic and hyperthermophilic microorganisms from hydrothermal vents and hydrothermal seep sediments[5,6,8,22,40,45] and the discovery of new catabolic pathways[13,23], and have expanded the known growth temperature ranges of microorganisms[18,19,22]. Many of the thermo- and hyperthermophiles isolated occur in both hydrothermal seep sediments and nearby hydrothermal vent chimneys[9,24], frequently even in samples that also harbor microorganisms with much lower temperature requirements[11,24]. Guaymas Basin sediment has also remained a treasure trove of novel microbial diversity. Gene sequences of the alpha subunit of methyl coenzyme M reductase (*mcrA*) indicate the presence of at least three novel candidate orders of

methane-cycling Archaea[4,24]. Recent genomic investigations have resulted in the proposal of 5 new candidate phyla within the Bacteria and Archaea[14]. *In situ* fluid geochemistry and temperature, and microbial resilience to rapidly fluctuating thermal regimes, have been proposed as key drivers of microbial community structure[12,32,34,46]. In addition, it has been proposed that high rates of thermal disturbance result in refugia for temperature-resilient microorganisms that are competitively excluded in more stable environments[24]. The existence of such refugia, combined with the fact that Guaymas Basin sediment unites characteristics of hydrothermal vents, oil reservoirs, and hydrocarbon seeps may further contribute to the astounding microbial diversity[24].

Despite the impressive number of past microbiological investigations, many questions concerning the controls on microbial community structure in Guaymas Basin sediment remain open. For instance, only few studies have investigated *in situ* microbial abundances, or their relationships with temperature[10,34]. Recent metagenomic data from 11 samples indicate that Archaea dominate hydrothermal sites[14], providing support to the hypothesis that Archaea are better equipped for life at high temperatures than Bacteria because of higher temperature resistance of archaeal ether-based lipid membranes compared to bacterial ester-based lipid membranes[48].

Most microbiological research on Guaymas Basin has so far focused on a hydrothermal field with hydrothermal vents in the Southern Trough (here referred to as Seep Area (SA)). Subseafloor fluid circulation through the surrounding sediment leads to the development of a hot, heterogeneous methane, sulfide, and petroleum seep area, characterized by the presence of *Beggiatoaceae* mats, hydrothermal mounds, and bare sediments[47]. Additional research has been performed on cold methane and CO_2 seeps at the northeastern transform margin (Sonora Margin)[9,37,49]. In 2015, a second hydrothermal vent field (here referred to as Non-Seep Area (NSA)) was discovered on the flank of the Northern Trough during Expedition SO241 of the research vessel SONNE[2]. Near this vent field, extensive authigenic carbonate crusts are present in the absence of clear fluid flow, indicating a previously active hydrothermal and cold seep environment, in which deep fluid and thermogenic gas flow terminated 7-28,000 kyrs ago[16]. Even though present-day vertical fluid advection was not detected at locations sampled during SO241, sites surrounding the vent field maintain significant temperature gradients, reaching temperatures of 60-70°C within 400-500 centimeters below the seafloor (mbsf)[2,16]. Due to the absence of new energy inputs from photosynthetically produced OM or hydrothermal fluid advection, these geothermally heated

non-seep sites offer the opportunity to investigate the role of temperature in driving microbial community structure under diffusion-controlled subseafloor conditions, where microorganisms are severely energy-limited.

Supplementary Methods

DNA extraction

DNA was extracted according to Lever *et al.* (2015) (reference 25). Briefly, all samples were extracted using lysis protocol II with the following specifications: 0.2 g sediment were placed into screw-cap microcentrifuge tubes filled to ~15% with 0.1mm zirconium-silica beads and mixed with 100 μL of 10mM sodium hexametaphosphate solution. Samples were homogenized for 30s at 30 shakings per second on a Tissue Lyzer LT (Qiagen). Afterward, chemical lysis for 1 hour at 50°C and 600 rpm was performed on a ThermoMixer (Eppendorf). Samples were then washed two times with ice-cold chloroform-isoamlyalcohol (24:1), and precipitated at room temperature in the dark with ethanol-sodium chloride solution supplemented with linear polyacrylamide (LPA) as a co-precipitant (20 µg LPA mL^{-1} extract). Precipitated and dried DNA pellets were purified using the CleanAll DNA/RNA Clean-Up and Concentration Micro Kit (Norgen Biotek, Madison, WI) according to the manufacturer's instructions. For further details on Lysis Protocol II, including all instrument settings, see Lever *et al.* (2015).

16S rRNA gene quantification

Bacterial and archaeal 16S rRNA gene copy numbers were quantified by SYBR-Green-based quantitative PCR (qPCR) on a LightCycler 480 Instrument II (Roche Life Science, Penzberg, Germany). The Bac908F_mod (5´- AAC TCA AAK GAA TTG ACG GG-3')[25] / Bac1075R (5´- CAC GAG CTG ACG ACA RCC-3´)[36] primer combination was used for Bacteria. The Arch915F_mod (5´-AAT TGG CGG GGG AGC AC-3´)[7] / Arch1059R (5´-GCC ATG CAC CWC CTC T-3´)[51] was used for Archaea. qPCR reactions (final volume: 10 µL) consisted of 5 μL 2 × SYBR Green I Master (Roche Life Science, Penzberg, Germany), 1 µg μL^{-1} bovine serum albumin, 10 µM of each primer, molecular-grade water, and 2 µL of original DNA extract. Amplicons of 16S rRNA genes of *Holophaga foetida* and *Thermoplasma acidophilum* were used as bacterial and archaeal qPCR standards. Pure cultures of *H. foetida* and *T. acidophilum* were purchased from the German Collection of Microorganisms and Cell Cultures and their DNA extracted using a MOBIO PowerSoil DNA

Isolation Kit (QIAGEN, Hilden, Germany. Thermal cycler protocols consisted of (1) enzyme activation and initial denaturation at 95 °C for 5 min; (2) 40 cycles (Bacteria) and 50 cycles (Archaea) of (a) denaturation at 95°C for 10 s, (b) annealing at 60°C (Bacteria) and 55°C (Archaea) for 30 s, (c) elongation at 72°C for 15 s, and (d) fluorescence measurement at 72°C (Bacteria) and 81°C (Archaea) for 15 s; and (3) a stepwise melting curve from 95°C to 55°C in 1 min to check for primer specificity. All measurements were run in duplicate. Samples with on average >3 times higher values than extraction blanks were included in the manuscript.

16S rRNA amplicon sequencing and phylogenetic classifications

According to 16S rRNA gene abundances, samples were pooled in different groups for a first booster PCR, performed with the aim of increasing and normalizing the gene copy number. The archaeal primer pair S-D-Arch-0519-a-A-19 (5'-C AGC MGC CGC GGT AAH ACC-3'; reference 43, renamed in reference 21) / Arch915RRmod (5'-GT GCT CCC CCG CCA ATT-3')[7] and the bacterial primer pair S-D-Bact-0341-b-S-17 (5'-CCT ACG GGN GGC WGC AG-3') / S-D-Bact-0785-a-A-21 (5'-GAC TAC HVG GGT ATC TAA TCC-3'; both reference 17, renamed in reference 21) were used for this booster PCR. Booster PCRs were used to elevate gene copy numbers to similar concentrations across samples and to minimize PCR cycle numbers with tailed primers, which introduce additional biases beyond those of non-tailed primers[3]. According to the original 16S gene copy numbers determined by qPCR, booster PCR cycle numbers varied from 10 to 32. PCR products of booster PCR were checked on an agarose gel, and those samples with visible (preferably weakly visible) correctly-sized bands were used for downstream work. Booster PCRs were repeated on samples with no visible bands, increasing the cycle number by 4 additional cycles. 1μL of PCR product from all successful booster PCRs was used as template for a second PCR (8 cycles) with frameshifted tailed primers, that were used to improve sequencing accuracy[29]. Tailed amplicons were then cleaned using AMPure XP beads, amplicon lengths checked by gel electrophoresis to confirm successful addition of adaptor (tail) sequences, and then underwent Index-PCR using the Nextera DNA library Prep Kit (Illumina, San Diego, USA). Indexed amplicons were cleaned using AMPure XP beads, quantified with a Tecan plate reader and Tapestation (Agilent, Santa Clara, USA), and finally pooled equimolarly. Paired-end sequencing (2x300 bp) was performed on a MiSeq Personal Sequencer (Illumina, San Diego, USA). Raw-read ends were trimmed and pairs merged into amplicons. Subsequently primers were trimmed and amplicons were quality filtered (PRINSEQ). Operational

taxonomic units (OTU) at 97% clustering were assigned using UNOISE. Taxonomic assignments were performed using the SILVA database (SSURef v128) for Bacteria and a manually curated in-house archaeal 16S rRNA gene database in ARB[28]. Sequencing data were analyzed using Phyloseq package[33]. Statistical analyses were perfomed in R using Vegan, heatmaps using package Corrplot[50].

Supplementary Figures

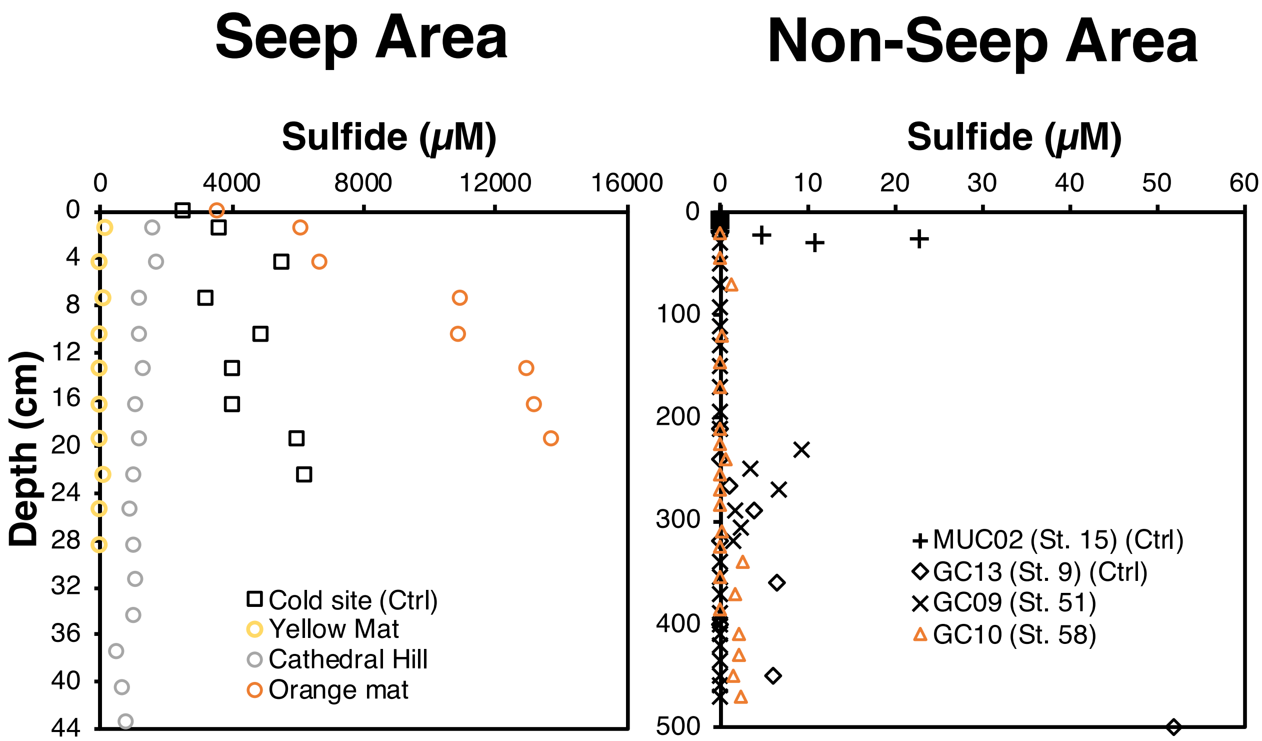

Supplementary Figure 1. Hydrogen sulfide concentration profiles in the Seep Area (SA) and Non-Seep Area (NSA) replotted from Reference 47 (SA) and Reference 16 (NSA; also see Supplementary Data 1). Note: no data exist for Everest Mound and MUC12.

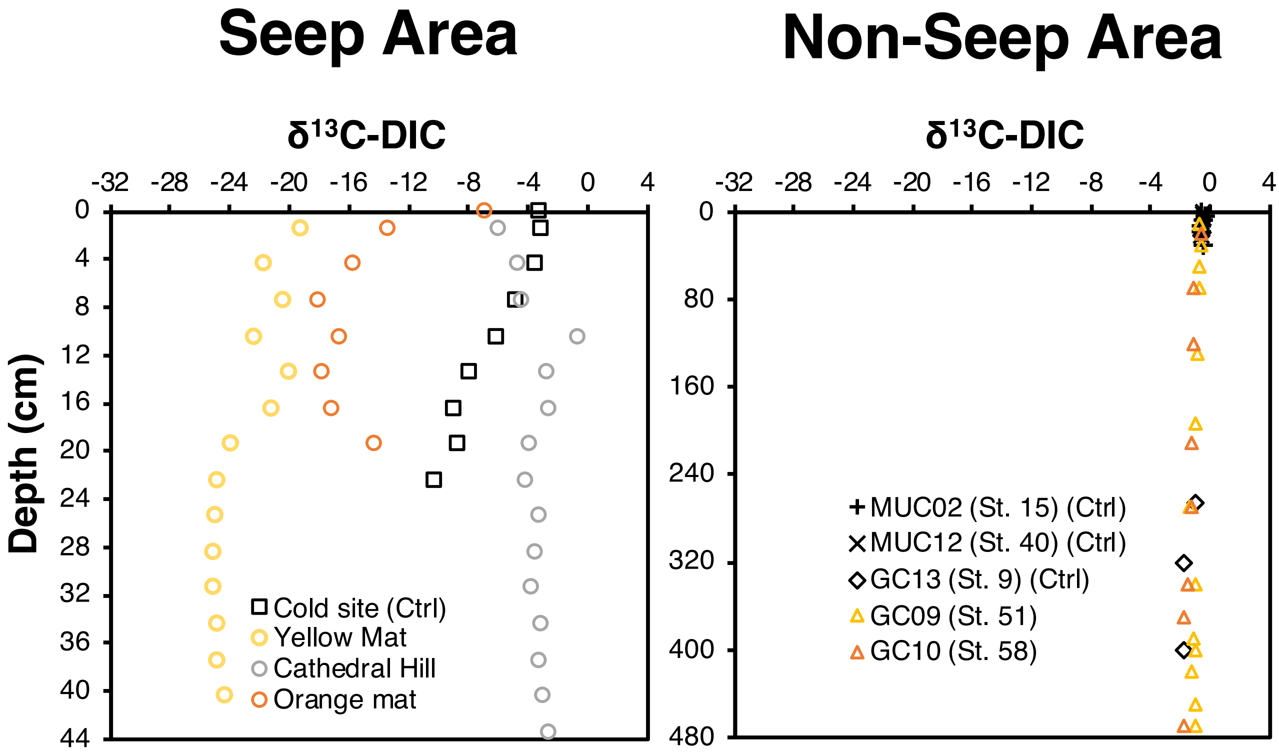

Supplementary Figure 2. Depth profiles δ^{13}C-dissolved inorganic carbon (δ^{13}C-DIC) in the Seep Area (SA) and Non-Seep Area (NSA). All data shown in Supplementary Data 1.

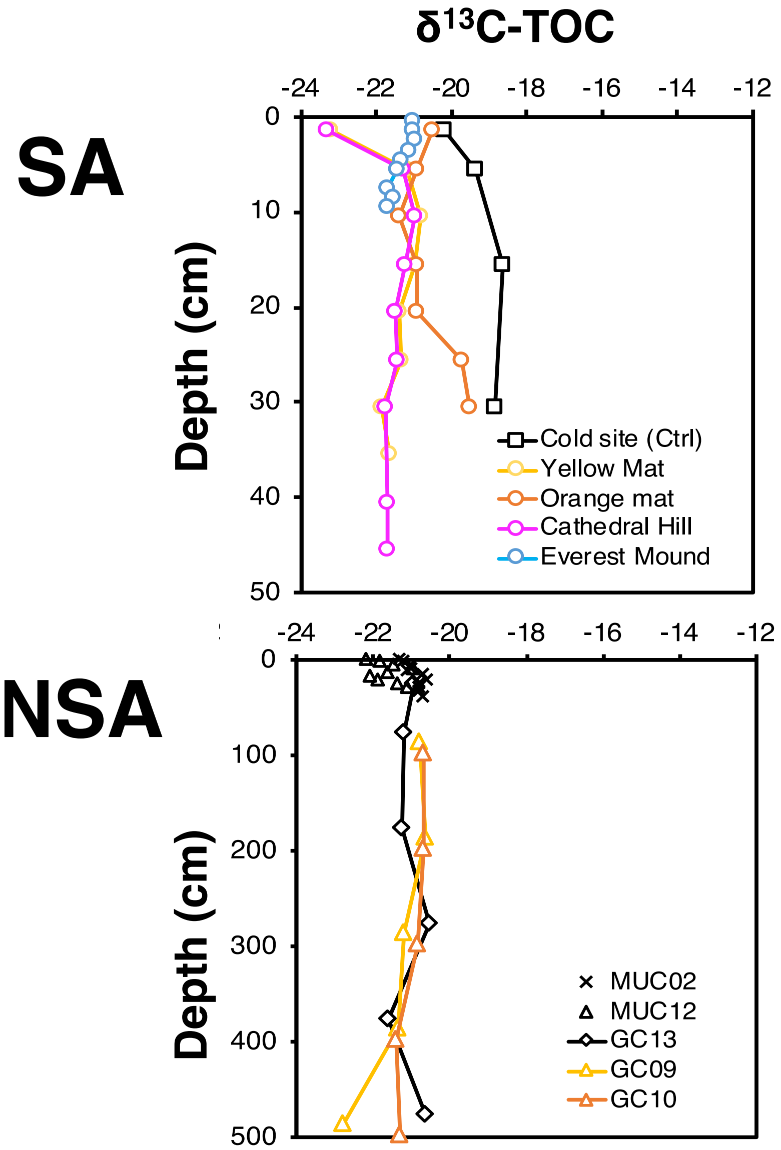

Supplementary Figure 3. Depth profiles of δ^{13}C-Total Organic Carbon (δ^{13}C-TOC) in the SA and NSA. All data shown in Supplementary Data 1.

Supplementary Figure S4A, Non-Seep Area (NSA)

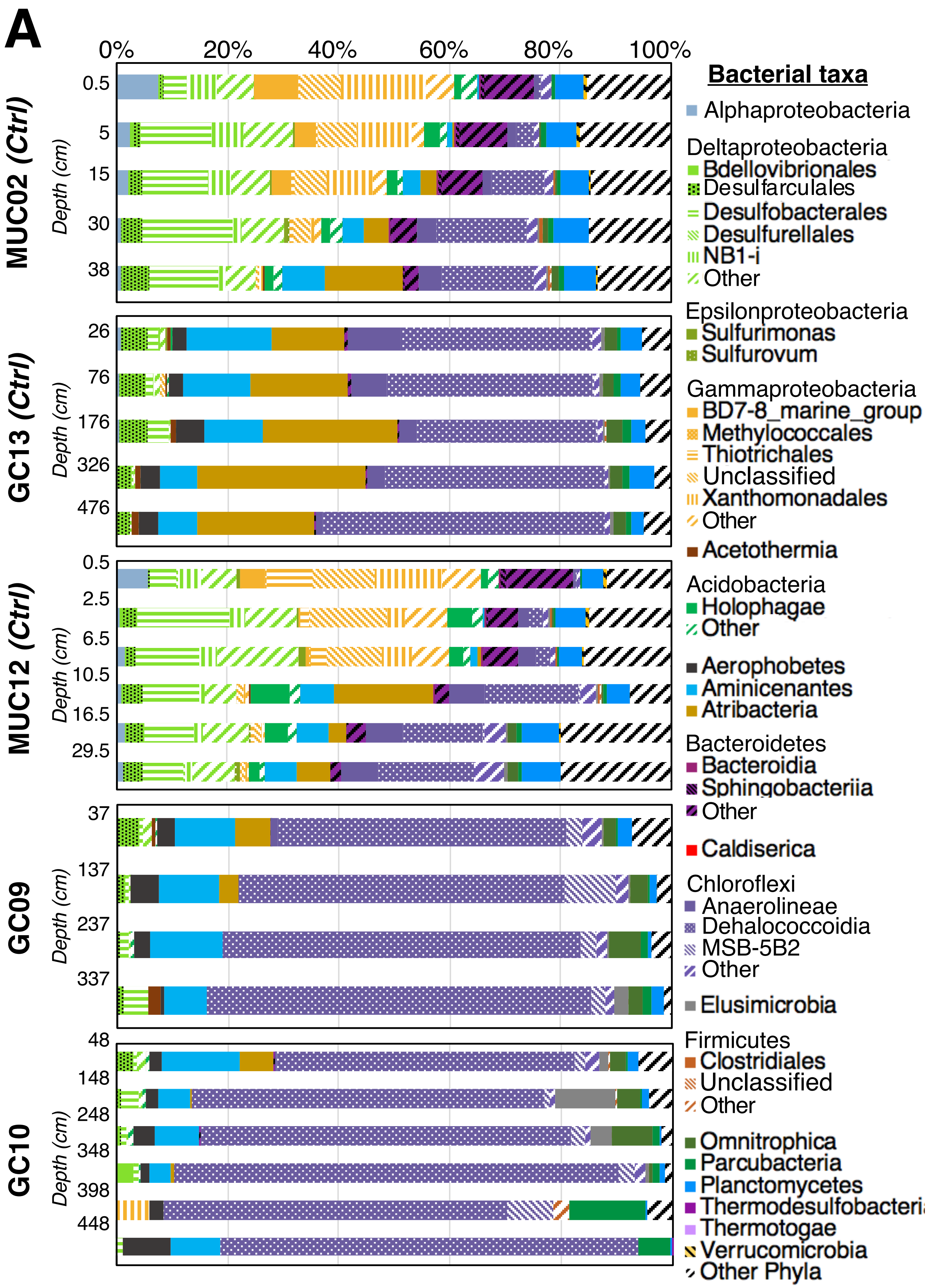

Supplementary Figure S4B, Seep Area (SA)

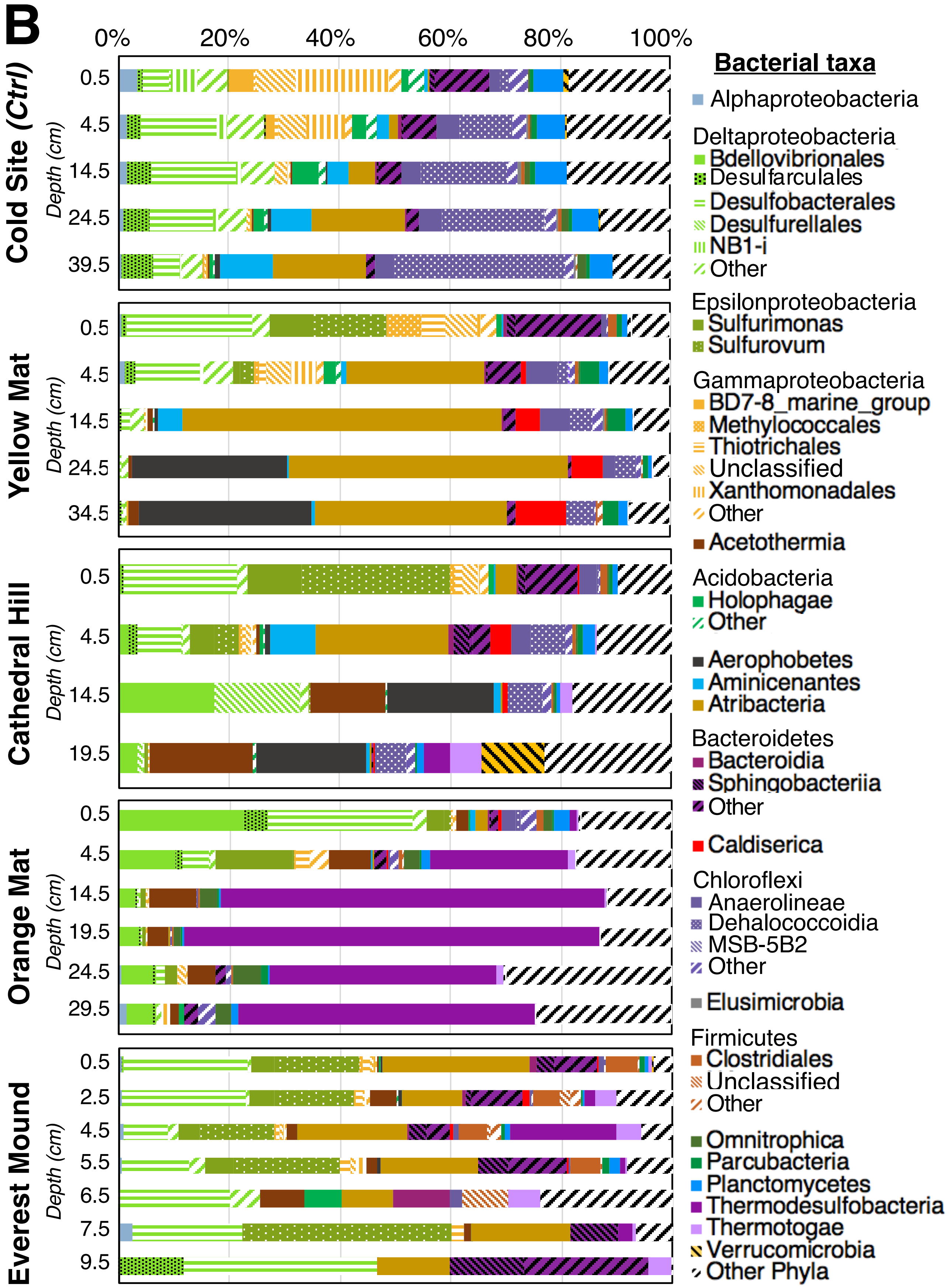

Supplementary Figure 4. Detailed depth profiles of bacterial community structure in the (a) NSA and (b) SA, focusing on the dominant classes, orders, and genera for phyla with sub-phylum classifications.

Supplementary Figure S5A, Non-Seep Area (NSA)

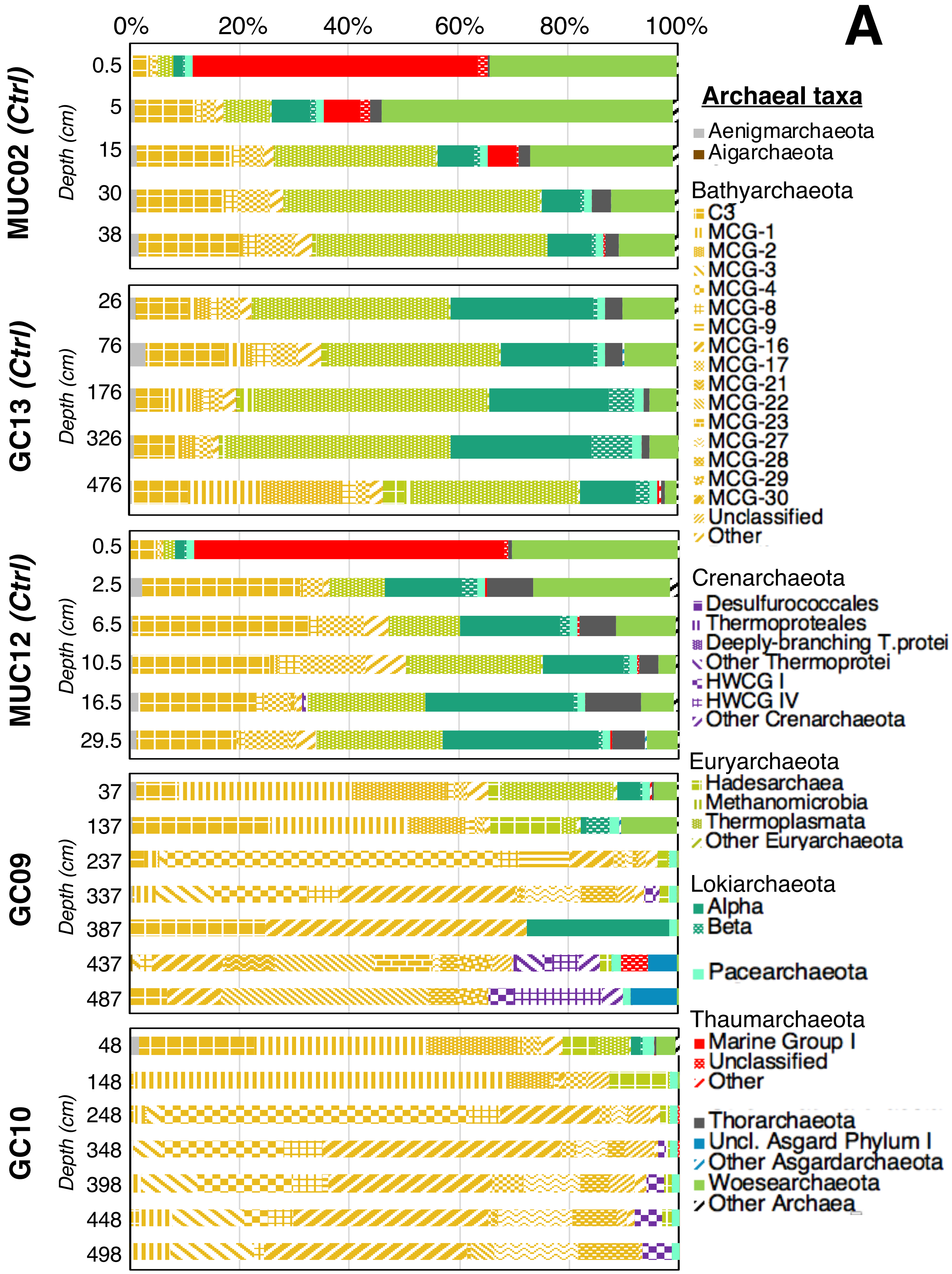

Supplementary Figure S5B, Non-Seep Area (NSA)

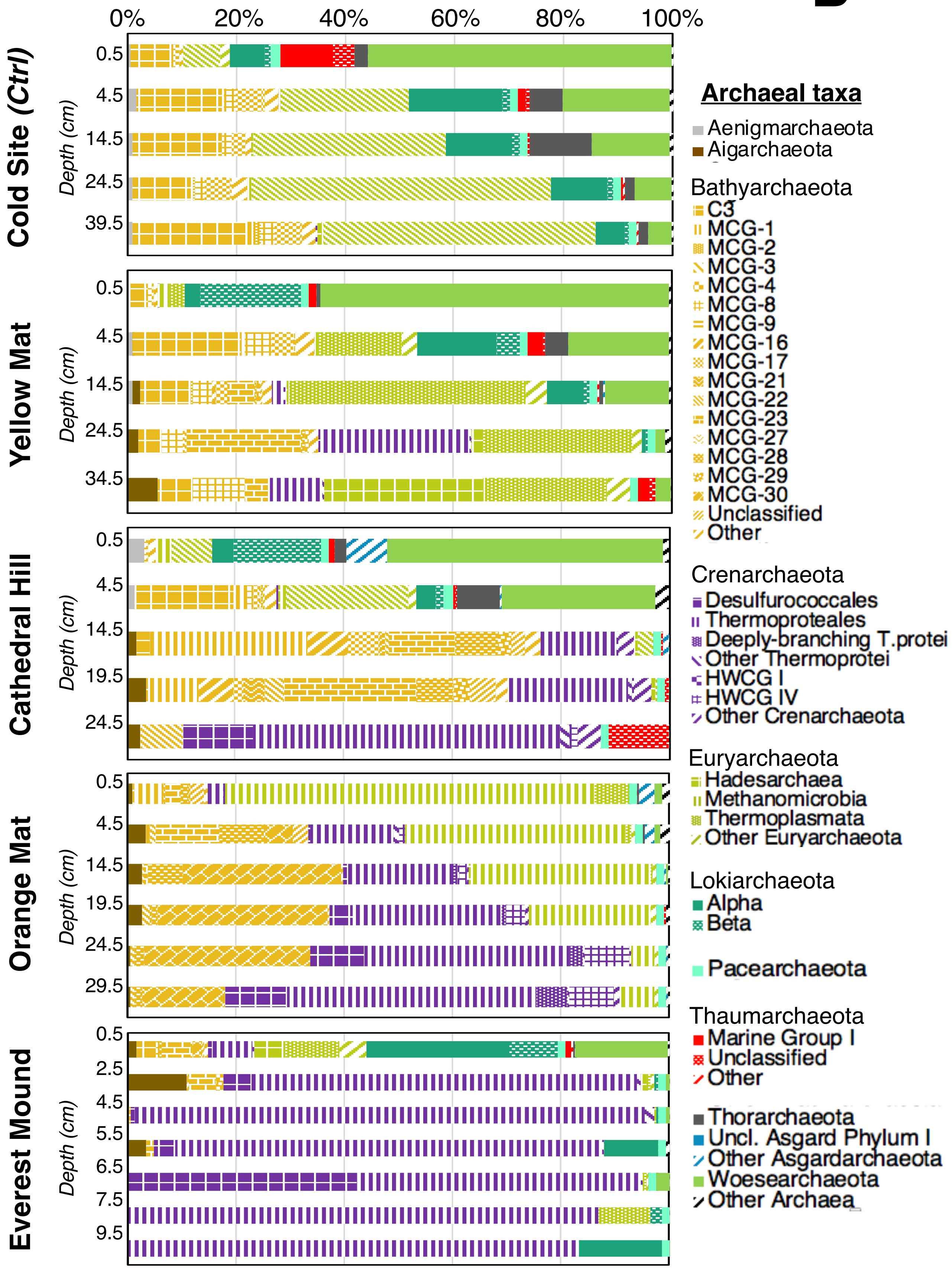

Supplementary Figure 5. Detailed depth profiles of archaeal community structure in the (a) NSA and (b) SA, focusing on the dominant classes, orders, and genera for phyla with sub-phylum classifications.

Supplementary Figure S6A, Cren- and Aigarchaeota

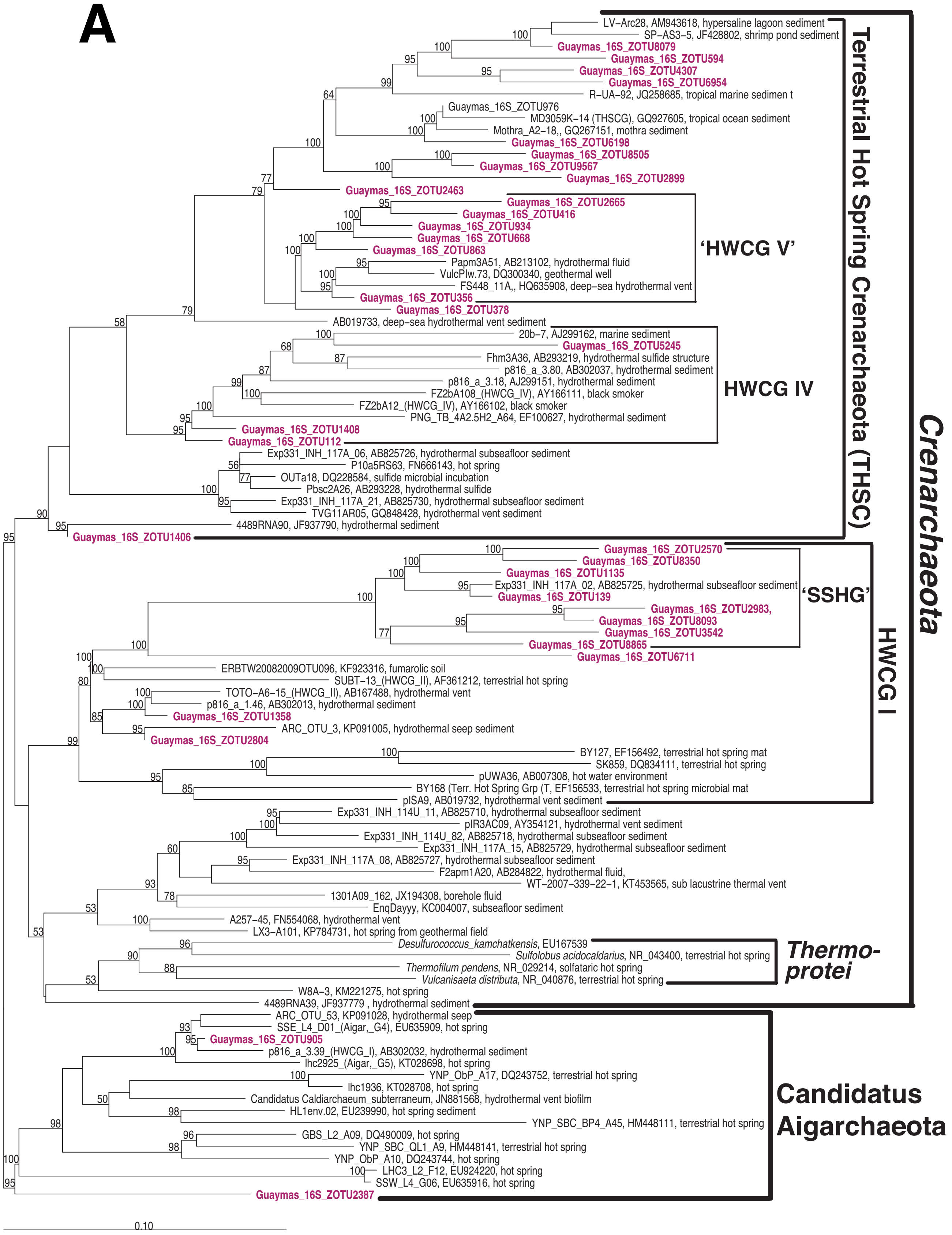

Supplementary Figure S6B, Crenarchaeotal Class Thermoprotei only

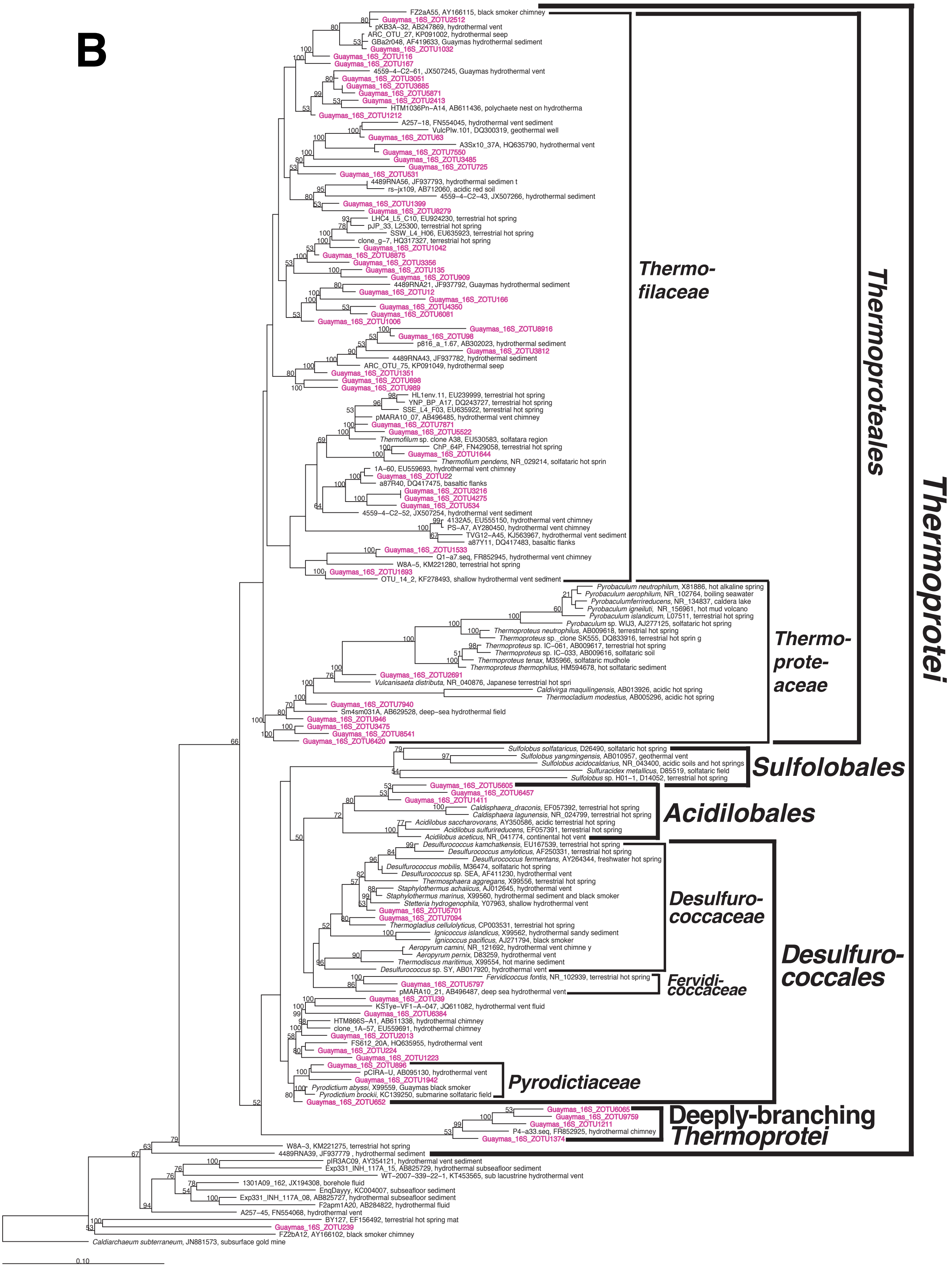

Previous 2 pages

Supplementary Figure 6. Phylogenetic trees of (a) Terrestrial Hot Spring Crenarchaeota (THSC) and Hot Water Crenarchaeote Group I (HWCG I) and *Aigarchaeota*, and (b) the crenarchaeotal class *Thermoprotei*. Trees are based on a manually optimized archaeal 16S rRNA gene alignment and were constructed by ARB Neighbor-Joining using Jukes-Cantor Correction combined with a column filter that excluded insertions and hypervariable regions. Subsurface Hydrothermal Group (SSHG) and Deeply-Branching *Thermoprotei* are newly classified groups within the HWCG I and *Thermoprotei*, respectively. Bootstrap values (1,000 repetitions) of ≥50% are shown at branch nodes.

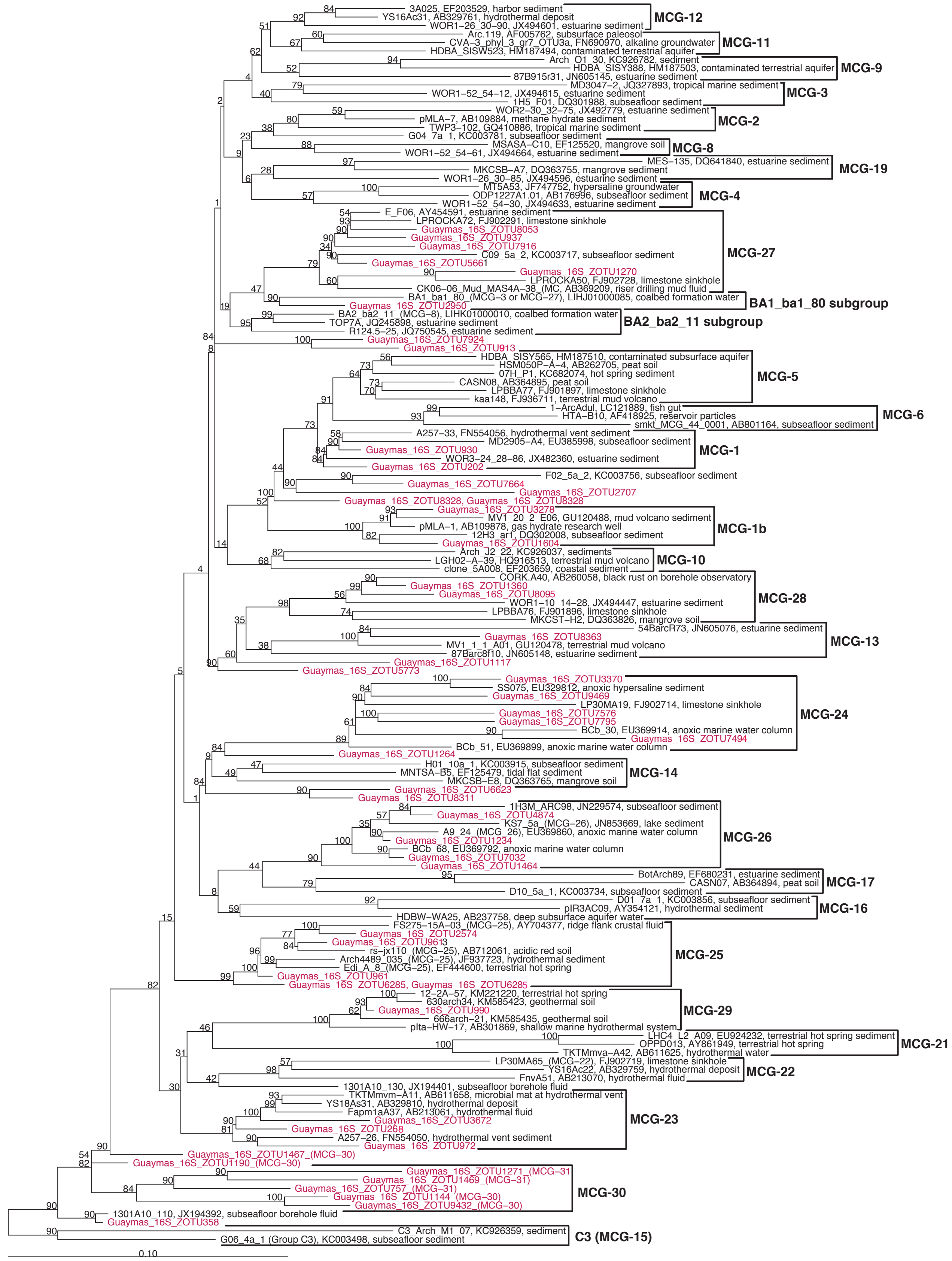

Supplementary Figure 7. Phylogenetic tree highlighting novel *Bathyarchaeota* subgroups (MCG-24 through -30), along with their closest relatives. The tree was produced by ARB Neighbor-Joining using Jukes-Cantor Correction and is based on a manually optimized archaeal 16S rRNA gene alignment with a column filter to exclude insertions and hypervariable regions. Note: C3 are also known as MCG-15. Bootstrap values (1,000 repetitions) of ≥50% are indicated at branch nodes.

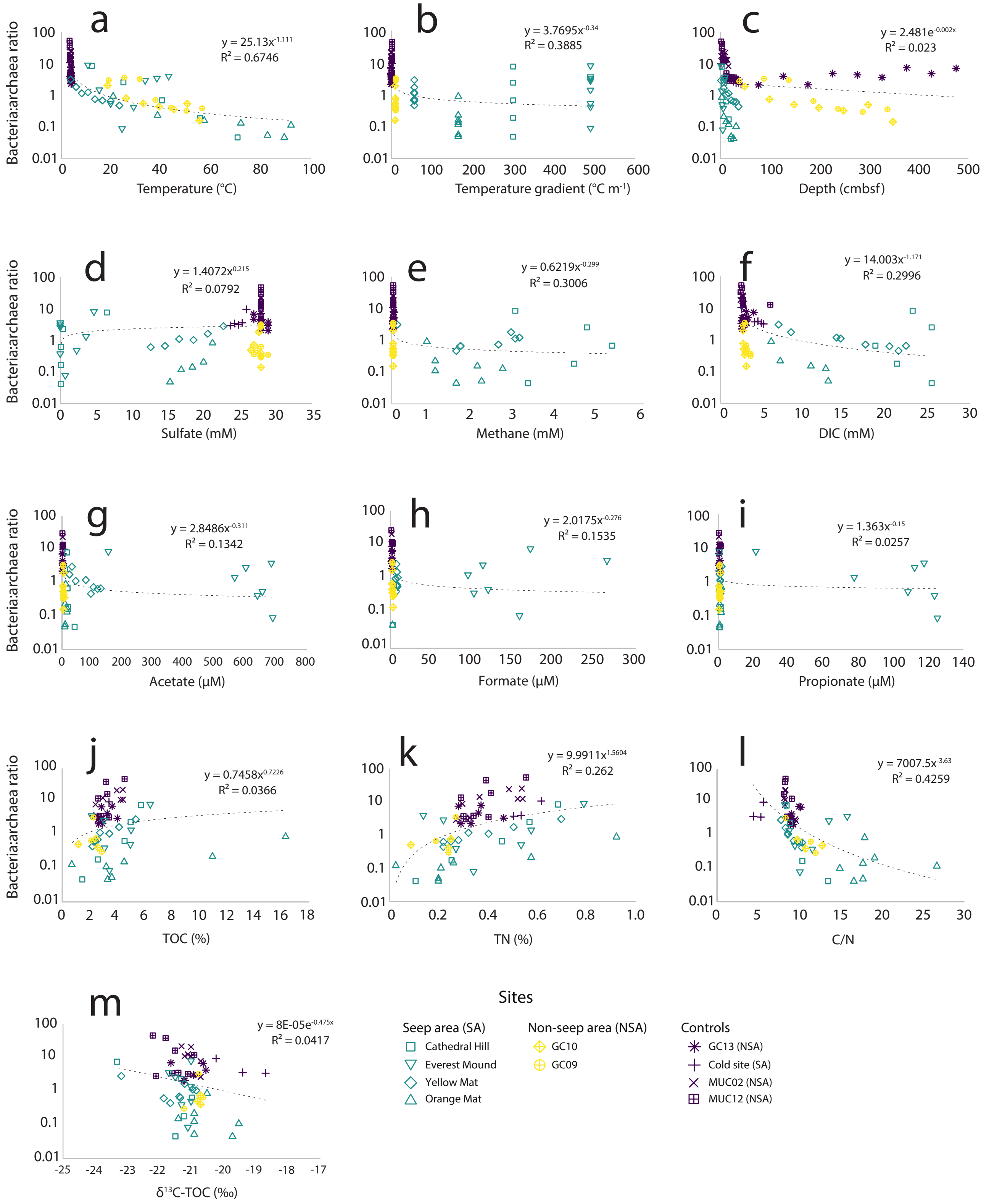

Supplementary Figure 8. Relationships between BARs and environmental variables. Environmental variables include (a) temperature, (b) temperature gradient, and (c) sediment depth, concentrations of (d) sulfate, (e) methane, (f) dissolved inorganic carbon (DIC), (g) acetate, (h) formate, and (i) propionate, as well as (j) total organic carbon (TOC), (k) total nitrogen (TN), (l) TOC/TN (C/N), and (m) δ^{13}C-TOC. Trendlines reflect best-fit functions in Microsoft Excel, which was also used to calculate coefficient of determination (R^2) values.

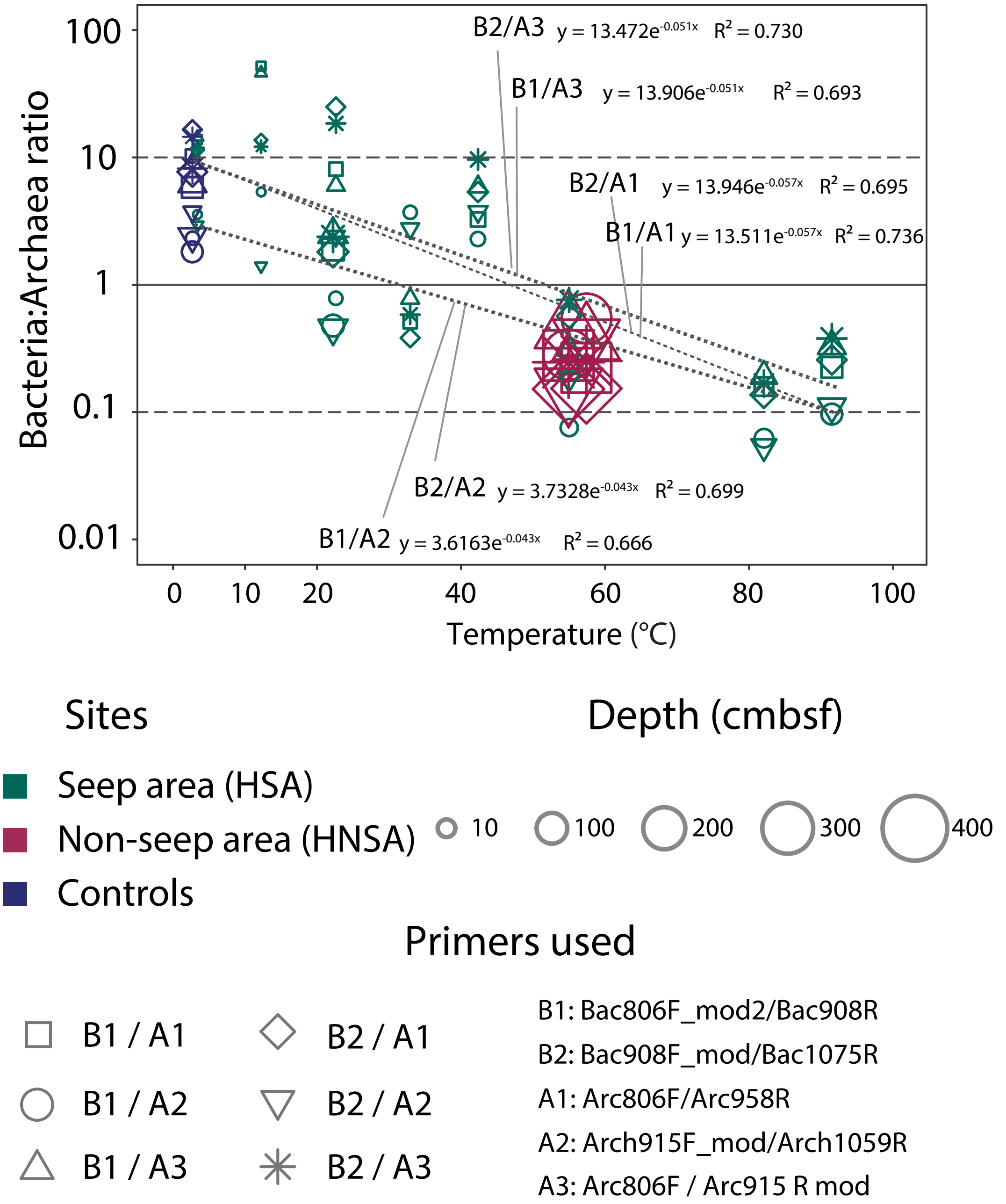

Supplementary Figure 9. Bacteria-to-Archaea 16S rRNA gene copy ratios (BARs) vs. temperature. Calculated for different bacterial and archaeal qPCR primer combinations on a subset of samples. The best-fit trendline in all cases follows an exponential function that was fitted in Microsoft Excel, which was also used to calculate the coefficient of determination (R^2) values. Results obtained with the primer pairs B2 and A2 on all samples are shown in Figures 1 and 2 and in Supplementary Figure S9.

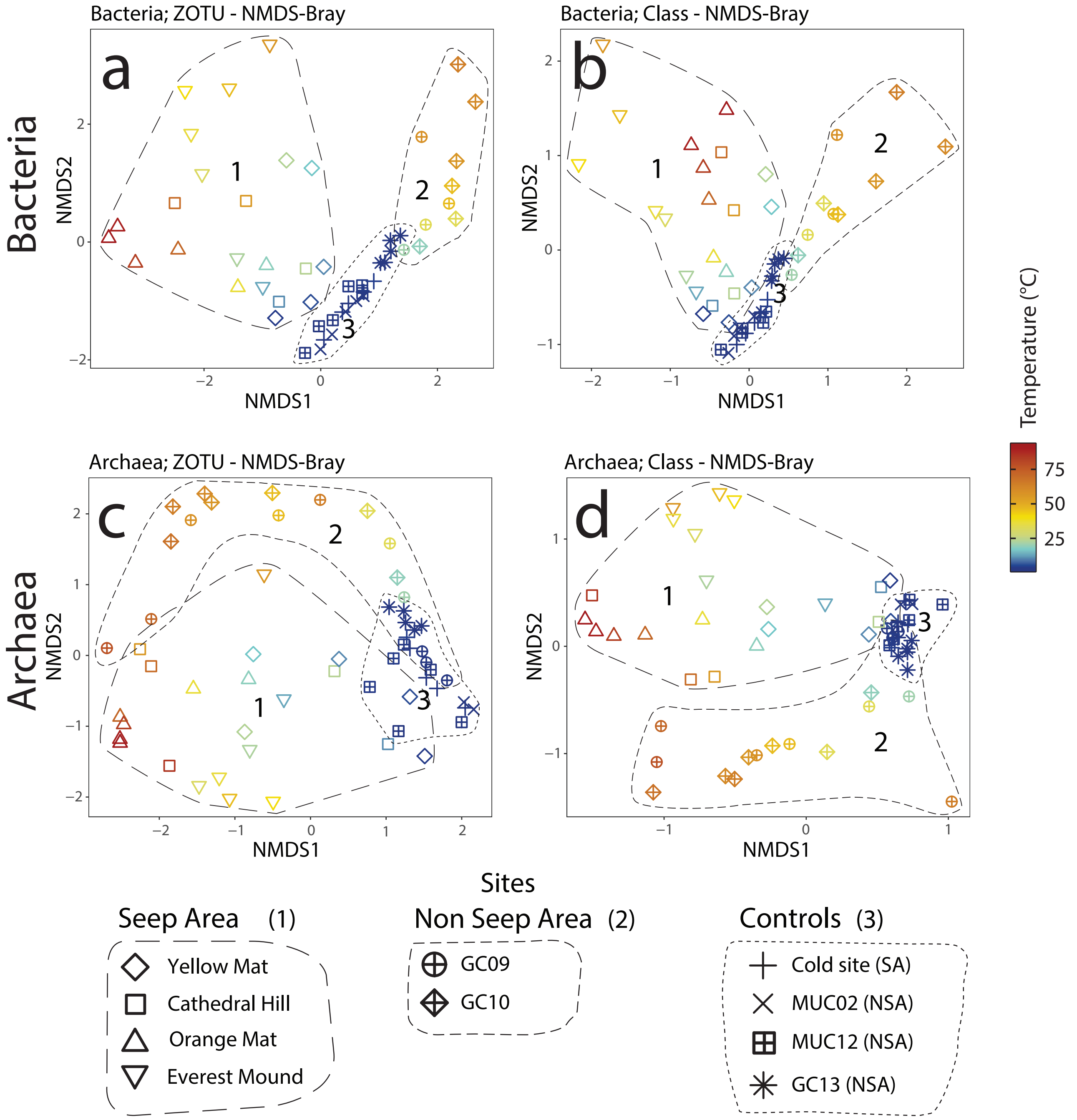

Supplementary Figure 10. Non-metric multi-dimensional scaling (NMDS) plots calculated with Bray-Curtis algorithms of ZOTU- and class-level bacterial and archaeal community structure across all sites. Cold control sites from both locations are grouped together in the legend for easier viewing.

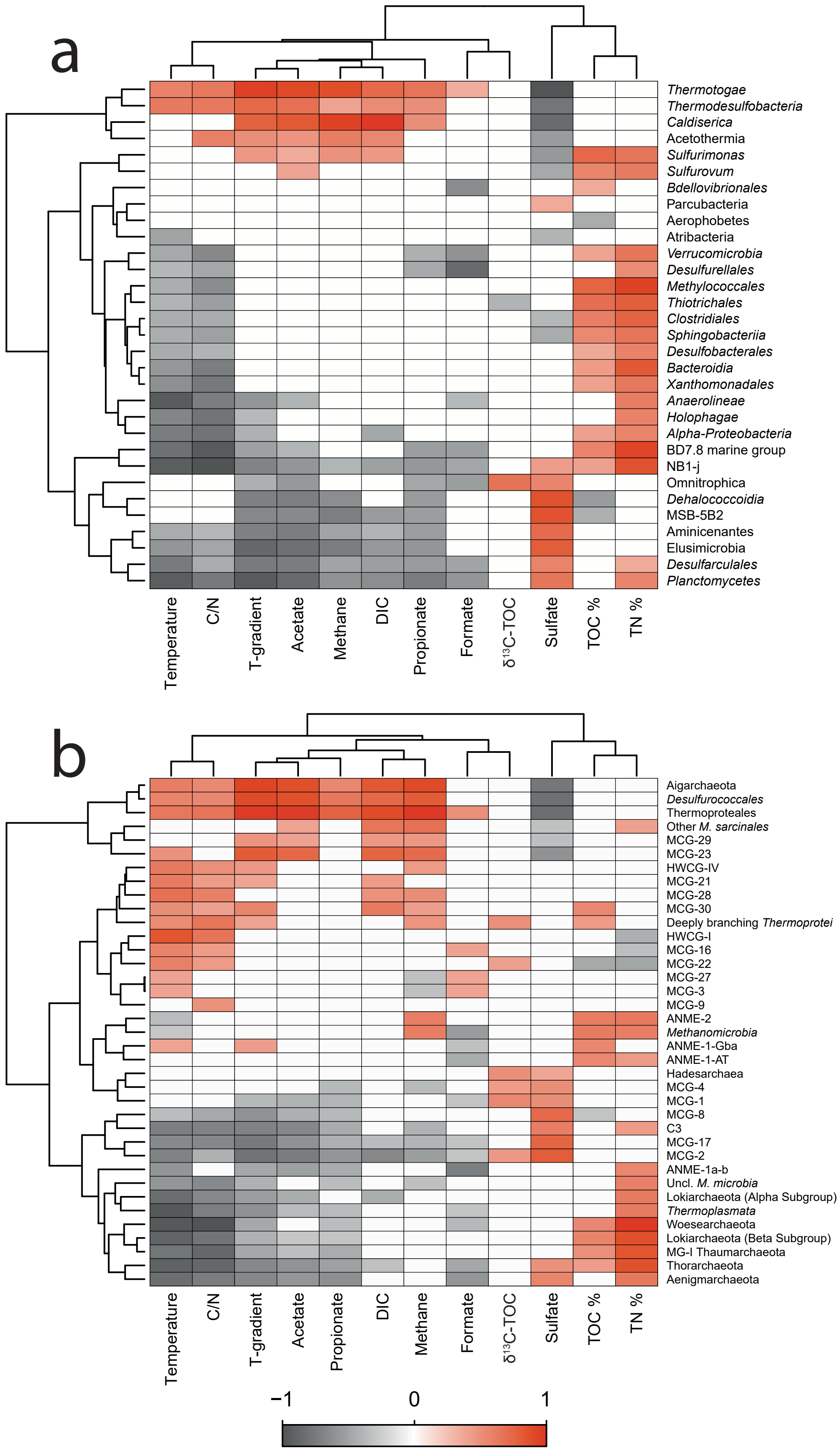

Supplementary Figure 11. Heat map showing correlations between relative abundances of key bacterial and archaeal groups and geochemical variables based on Spearman's rank correlation coefficients. Only significant ($p<0.05$) correlations are colored.

Supplementary References

(1) Bazylinski DA, Farrington JW, Jannasch HW. Hydrocarbons in surface sediments from a Guaymas Basin hydrothermal vent site. *Org Geochem* 1988; **12:** 547-558.

(2) Berndt C, Hensen C, Mortera-Gutierrez C, Sarkar S, Geilert S, Schmidt M, *et al.* Rifting under steam—How rift magmatism triggers methane venting from sedimentary basins. *Geology* 2016; **44**: 767-770.

(3) Berry D, Ben Mahfoudh K, Wagner M, Loy A. Barcoded primers used in multiplex amplicon pyrosequencing bias amplification. *Appl Environ Microbiol* 2011; **77**: 7846-7849.

(4) Biddle JF, Cardman Z, Mendlovitz H, Albert DB, Lloyd KG, Boetius A, *et al.* Anaerobic oxidation of methane at different temperature regimes in Guaymas Basin hydrothermal sediments. *ISME J* 2012; **6**: 1018-1031.

(5) Burggraf S, Fricke H, Neuner A, Kristjansson J, Rouvier P, Mandelco L, *et al.* *Methanococcus igneus* sp. nov., a novel hyperthermophilic methanogen from a shallow submarine hydrothermal system. *Syst Appl Microbiol* 1990; **13**: 263-269.

(6) Burggraf S, Jannasch HW, Nicolaus B, Stetter KO. *Archaeoglobus profundus* sp. nov. represents a new species within the sulfate-reducing Archaebacteria. *Syst Appl Microbiol* 1990; **13**: 24-28.

(7) Cadillo-Quiroz H, Brauer S, Yashiro E, Sun C, Yavitt J, Zinder S. Vertical profiles of methanogenesis and methanogens in two contrasting acidic peatlands in central New York State, USA. *Environm Microbiol* 2006; **8**: 1428-1440.

(8) Canganella F, Jones WJ, Gambacorta A, Antranikian G. *Thermococcus guaymasensis* sp. nov. and *Thermococcus aggregans* sp. nov., two novel thermophilic archaea isolated from the Guaymas Basin hydrothermal vent site. *Int J Syst Bacteriol* 1998; **48**: 1181-1185.

(9) Cruaud P, Vigneron A, Pignet P, Caprais J-C, Lesongeur F, Toffin L, *et al.* Comparative study of Guaymas Basin microbiomes: cold seeps vs. hydrothermal vents sediments. *Frontiers Mar Sci* 2017; **4**: 417.

(10) Deng LH, Fiskal A, Han XG, Dubois N, Bernasconi SM, Lever MA. Improving the accuracy of flow cytometric quantification of microbial populations in sediments: importance of cell staining procedures. *Frontiers Microbiol* 2019; **10**: 720.

(11) Dhillon A, Lever M, Lloyd KG, Albert DB, Sogin ML, Teske A. Methanogen diversity evidenced by molecular characterization of methyl coenzyme M reductase A (*mcrA*) genes in hydrothermal sediments of the Guaymas Basin. *Appl Environ Microbiol* 2005; **71**: 4592-4601.

(12) Dick GJ. The microbiomes of deep-sea hydrothermal vents: distributed globally, shaped locally. *Nature Rev Microbiol* 2019; **17**: 271-283.

(13) Dombrowski N, Seitz KW, Teske AP, Baker BJ. Genomic insights into potential interdependencies in microbial hydrocarbon and nutrient cycling in hydrothermal sediments. *Microbiome* 2017; **5**: 106.

(14) Dombrowski N, Teske AP, Baker BJ. Expansive microbial metabolic versatility and biodiversity in dynamic Guaymas Basin hydrothermal sediments. *Nature Comm* 2018; **9**: 4999.

(15) Einsele G, Gieskes JM, Curray J, Moore DM, Aguayo E, Aubry MP, *et al.* Intrusion of basaltic sills into highly porous sediments, and resulting hydrothermal activity. *Nature* 1980; **283**: 441-445.

(16) Geilert S, Hensen C, Schmidt M, Liebetrau V, Scholz F, Doll M, *et al.* On the formation of hydrothermal vents and cold seeps in the Guaymas Basin, Gulf of California. *Biogeosciences* 2018; **15**: 5715-5731.

(17) Herlemann DPR, Labrenz M, Jurgens K, Bertilsson S, Waniek JJ, Andersson AF. Transitions in bacterial communities along the 2000 km salinity gradient of the Baltic Sea. *ISME J* 2011; **5**: 1571-1579.

(18) Holler T, Widdel F, Knittel K, Amann R, Kellermann MY, Hinrichs KU, *et al.* Thermophilic anaerobic oxidation of methane by marine microbial consortia. *ISME J* 2011; **5**: 1946-1956.

(19) Jørgensen BB, Isaksen MF, Jannasch HW. Bacterial sulfate reduction above 100°C in deep-sea hydrothermal vent sediments. *Science* 1992; **258**: 1756-1757.

(20) Jørgensen BB, Boetius A. Feast and famine--microbial life in the deep-sea bed. *Nat Rev Microbiol* 2007; **5**: 770-781.

(21) Klindworth A, Pruesse E, Schweer T, Peplies J, Quast C, Horn M, *et al.* Evaluation of general 16S ribosomal RNA gene PCR primers for classical and next-generation sequencing-based diversity studies. *Nucl Acids Res* 2013; **41**: e1.

(22) Kurr M, Huber R, König H, Jannasch HW, Fricke H, Trincone A, *et al*. *Methanopyrus kandleri*, gen. and sp. nov. represents a novel group of hyperthermophilic methanogens, growing at 110°C. *Arch Microbiol* 1991; **156**: 239-247.

(23) Laso-Pérez R, Wegener G, Knittel K, Widdel F, Harding KJ, Krukenberg V, *et al.* Thermophilic archaea activate butane via alkyl-coenzyme M formation. *Nature* 2016; **539**: 396-401.

(24) Lever MA, Teske AP. Diversity of methane-cycling Archaea in hydrothermal sediment investigated by general and group-specific PCR primers. *Appl Environ Microbiol* 2015; **81**: 1426-1441.

(25) Lever MA, Torti A, Eickenbusch P, Michaud AB, Santl-Temkiv T, Jørgensen BB. A modular method for the extraction of DNA and RNA, and the separation of DNA pools from diverse environmental sample types. *Front Microbiol* 2015; **6**: 476.

(26) Lin YS, Koch BP, Feseker T, Ziervogel K, Goldhammer T, Schmidt F, *et al.* Near-surface heating of young rift sediment causes mass production and discharge of reactive dissolved organic matter. *Scient Repts* 2017*;* **7**: 44864.

(27) Lizarralde D, Soule SA, Seewald JS, Proskurowski G. Carbon release by off-axis magmatism in a young sedimented spreading centre. *Nature Geosci* 2011; **4**: 50-54.

(28) Ludwig W, Strunk O, Westram R, Richter L, Meier H, Yadhukumar, *et al.* ARB: a software environment for sequence data. *Nucl Acids Res* 2004; **32**: 1363-1371.

(29) Lundberg DS, Yourstone S, Mieczkowski P, Jones CD, Dangl JL. Practical innovations for high-throughput amplicon sequencing. *Nat Methods* 2013; **10**: 999-1002.

(30) Martens CS. Generation of short chain organic-acid anions in hydrothermally altered sediments of the Guaymas Basin, Gulf of California. *Appl Geochem* 1990; **5**: 71-76.

(31) McKay LJ, MacGregor BJ, Biddle JF, Albert DB, Mendlovitz HP, Hoer DR, *et al.* Spatial heterogeneity and underlying geochemistry of phylogenetically diverse orange and white Beggiatoa mats in Guaymas Basin hydrothermal sediments. *Deep Sea Research Part I* 2012; **67**: 21-31.

(32) McKay L, Klokman VW, Mendlovitz HP, LaRowe DE, Hoer DR, Albert D, *et al.* Thermal and geochemical influences on microbial biogeography in the hydrothermal sediments of Guaymas Basin, Gulf of California. *Environ Microbiol Repts* 2016; **8**: 150-161.

(33) McMurdie PJ, Holmes S. Phyloseq: an R package for reproducible interactive analysis and graphics of microbiome census data. *PloS ONE* 2013; **8**: e61217.

(34) Meyer S, Wegener G, Lloyd KG, Teske A, Boetius A, Ramette A. Microbial habitat connectivity across spatial scales and hydrothermal temperature gradients at Guaymas Basin. *Front Microbiol* 2013; **4**: 207.

(35) Nelson DC, Wirsen CO, Jannasch HW. Characterization of large, autotrophic *Beggiatoa spp.* abundant at hydrothermal vents of the Guaymas Basin. *Appl Environ Microbiol* 1989; **55**: 2909-2917.

(36) Ohkuma M, Kudo T. Phylogenetic analysis of the symbiotic intestinal microflora of the termite *Cryptotermes domesticus*. *FEMS Microbiol Lett* 1998; **164**: 389-395.

(37) Paull CK, Ussler W, Peltzer ET, Brewer PG, Keaten R, Mitts PJ, *et al.* Authigenic carbon entombed in methane-soaked sediments from the northeastern transform margin of the Guaymas Basin, Gulf of California. *Deep-Sea Res Part II* 2007; **54**: 1240-1267.

(38) Pearson A, Seewald JS, Eglinton TI. Bacterial incorporation of relict carbon in the hydrothermal environment of Guaymas Basin. *Geochim Cosmochim Acta* 2005; **69**: 5477-5486.

(39) Portail M, Olu K, Dubois SF, Escobar-Briones E, Gelinas Y, Menot L, Sarrazin J. Food-web complexity in Guaymas Basin hydrothermal vents and cold seeps. *PLoS One* 2016; **11**: e0162263.

(40) Reysenbach AL, Liu YT, Banta AB, Beveridge TJ, Kirshtein JD, Schouten S, *et al.* (2006). A ubiquitous thermoacidophilic archaeon from deep-sea hydrothermal vents. *Nature* 2006; **442**: 444-447.

(41) Schutte CA, Teske A, MacGregor BJ, Salman-Carvalho V, Lavik G, Hach P, de Beer D. Filamentous giant *Beggiatoaceae* from the Guaymas Basin are capable of both denitrification and dissimilatory nitrate reduction to ammonium. *Appl Environ Microbiol* 2018; **84**: e02860-17.

(42) Simoneit BRT, Lonsdale PF. Hydrothermal petroleum in mineralized mounds at the seabed of Guaymas Basin. *Nature* 1982; **295**: 198-202.

(43) Sørensen KB, Teske A. Stratified communities of active archaea in deep marine subsurface sediments. *Appl Environ Microbiol* 2006; **72**: 4596-4603.

(44) Teske A, Hinrichs KU, Edgcomb V, Gomez AD, Kysela D, Sylva SP, *et al.* Microbial diversity of hydrothermal sediments in the Guaymas Basin: Evidence for anaerobic methanotrophic communities. *Appl Environ Microbiol* 2002; **68**: 1994-2007.

(45) Teske A, Edgcomb V, Rivers AR, Thompson JR, Gomez AD, Molyneaux SJ, Wirsen CO. A molecular and physiological survey of a diverse collection of hydrothermal vent *Thermococcus* and *Pyrococcus* isolates. *Extremophiles* 2009; **13**: 905-915.

(46) Teske A, Callaghan AV, LaRowe DE. Biosphere frontiers of subsurface life in the sedimented hydrothermal system of Guaymas Basin. *Frontiers Microbiol* 2014; **5**: 362.

(47) Teske A, de Beer D, McKay LJ, Tivey MK, Biddle JF, Hoer D, *et al*. The Guaymas Basin hiking guide to hydrothermal mounds, chimneys, and microbial mats: complex seafloor expressions of subsurface hydrothermal circulation. *Frontiers Microbiol* 2016; **7**: 75.

(48) Valentine DL. Adaptations to energy stress dictate the ecology and evolution of the Archaea. *Nature Rev Microbiol* 2007; **5**: 316-323.

(49) Vigneron A, Cruaud P, Pignet P, Caprais JC, Cambon-Bonavita MA, Godfroy A, Toffin L. Archaeal and anaerobic methane oxidizer communities in the Sonora Margin cold seeps, Guaymas Basin (Gulf of California). *ISME J* 2013; **7**: 1595-1608.

(50) Wei T, Simko V. R package "corrplot": Visualization of a Correlation Matrix 2017; v. 0.84.

(51) Yu Y, Lee C, Kim J, Hwang S. Group-specific primer and probe sets to detect methanogenic communities using quantitative real-time polymerase chain reaction. *Biotechnol Bioeng* 2005; **89**: 670-679.
