# Supplementary material for: Interactions between temperature and energy supply drive microbial communities in hydrothermal sediment
Source: Commun Biol. 2021 Aug 25;4:1006. doi: 10.1038/s42003-021-02507-1 (PMC8387401; doi:10.1038/s42003-021-02507-1)
Supplement: Supplementary file 3 — Description of Additional Supplementary Files [file 42003_2021_2507_MOESM3_ESM.pdf]

Description of Additional Supplementary Files

File Name: Supplementary Data 1
Description: Overviews of all geochemical and quantitative PCR data.

File Name: Supplementary Data 2
Description: Phylogenetic summary tables of Bacteria, Archaea, and specifically methane-cycling archaea.

File Name: Supplementary Data 3
Description: Read contributions of phylogenetically annotated bacterial ZOTUs across all samples.

File Name: Supplementary Data 4
Description: Read contributions of phylogenetically annotated archaeal ZOTUs across all samples.
