# Supplementary material for: Interactions between temperature and energy supply drive microbial communities in hydrothermal sediment
Source: Commun Biol. 2021 Aug 25;4:1006. doi: 10.1038/s42003-021-02507-1 (PMC8387401; doi:10.1038/s42003-021-02507-1)
Supplement: Supplementary file 8 — Reporting Summary [file 42003_2021_2507_MOESM8_ESM.pdf]

nature research

Corresponding author(s): *Double-blind peer review submissions: write DBPR and your manuscript number here instead of author names.*

Last updated by author(s): *YYYY-MM-DD*

Reporting Summary

Nature Research wishes to improve the reproducibility of the work that we publish. This form provides structure for consistency and transparency in reporting. For further information on Nature Research policies, see our Editorial Policies and the Editorial Policy Checklist.

Statistics

For all statistical analyses, confirm that the following items are present in the figure legend, table legend, main text, or Methods section.

n/a	Confirmed	
☐	☒	The exact sample size (*n*) for each experimental group/condition, given as a discrete number and unit of measurement
☐	☒	A statement on whether measurements were taken from distinct samples or whether the same sample was measured repeatedly
☒	☐	The statistical test(s) used AND whether they are one- or two-sided<br>*Only common tests should be described solely by name; describe more complex techniques in the Methods section.*
☒	☐	A description of all covariates tested
☒	☐	A description of any assumptions or corrections, such as tests of normality and adjustment for multiple comparisons
☒	☐	A full description of the statistical parameters including central tendency (e.g. means) or other basic estimates (e.g. regression coefficient) AND variation (e.g. standard deviation) or associated estimates of uncertainty (e.g. confidence intervals)
☒	☐	For null hypothesis testing, the test statistic (e.g. *F*, *t*, *r*) with confidence intervals, effect sizes, degrees of freedom and *P* value noted<br>*Give P values as exact values whenever suitable.*
☒	☐	For Bayesian analysis, information on the choice of priors and Markov chain Monte Carlo settings
☐	☒	For hierarchical and complex designs, identification of the appropriate level for tests and full reporting of outcomes
☒	☐	Estimates of effect sizes (e.g. Cohen's *d*, Pearson's *r*), indicating how they were calculated

Our web collection on statistics for biologists contains articles on many of the points above.

Software and code

Policy information about availability of computer code

Data collection	Raw-read ends of 16S rRNA gene amplicons were trimmed and pairs merged into amplicons. Subsequently primers were trimmed and amplicons were quality filtered (PRINSEQ). Operational taxonomic units (OTU) at 97% clustering were assigned using UNOISE. Taxonomic assignments were performed using the SILVA database (SSURef v128) for Bacteria and a manually curated in-house archaeal 16S rRNA gene database in ARB. Sequencing data were analyzed using Phyloseq package. Statistical analyses were perfomed in R using Vegan, heatmaps using package Corrplot.
Data analysis	Raw-read ends of 16S rRNA gene amplicons were trimmed and pairs merged into amplicons. Subsequently primers were trimmed and amplicons were quality filtered (PRINSEQ). Operational taxonomic units (OTU) at 97% clustering were assigned using UNOISE. Taxonomic assignments were performed using the SILVA database (SSURef v128) for Bacteria and a manually curated in-house archaeal 16S rRNA gene database in ARB28. Sequencing data were analyzed using Phyloseq package. Statistical analyses were perfomed in R using Vegan, heatmaps using package Corrplot.

For manuscripts utilizing custom algorithms or software that are central to the research but not yet described in published literature, software must be made available to editors and reviewers. We strongly encourage code deposition in a community repository (e.g. GitHub). See the Nature Research guidelines for submitting code & software for further information.

Data

Policy information about availability of data

All manuscripts must include a data availability statement. This statement should provide the following information, where applicable:

- Accession codes, unique identifiers, or web links for publicly available datasets
- A list of figures that have associated raw data
- A description of any restrictions on data availability

Zero-noise operational taxonomic units (ZOTUs; 97% clustering) of 16S rRNA genes were generated using UNOISE and can be retrieved from the National Center for Biotechnology Information website under accession no. KDPV00000000. All geochemical data and phylogenetic assignments are included as supplementary data files with the manuscript.

Field-specific reporting

Please select the one below that is the best fit for your research. If you are not sure, read the appropriate sections before making your selection.

☐ Life sciences ☐ Behavioural & social sciences ☒ Ecological, evolutionary & environmental sciences

For a reference copy of the document with all sections, see nature.com/documents/nr-reporting-summary-flat.pdf

Ecological, evolutionary & environmental sciences study design

All studies must disclose on these points even when the disclosure is negative.

Study description	Temperature and bioavailable energy control the distribution of life on Earth, and interact with each other due to the dependency of biological energy requirements on temperature. Here we analyze how temperature-energy interactions structure sediment microbial communities in two hydrothermally active areas of Guaymas Basin.
Research sample	Marine sediment samples from Guaymas Basin (Gulf of California) were used for DNA-based microbiological analyses and a wide range of geochemical and stable isotopic analyses.
Sampling strategy	All samples were obtained by sediment coring. A total of 10 sites was analyzed. 8-10 samples were analyzed across each core, to provide a detailed record of vertical changes in microbial communites and geochemical gradients
Data collection	We performed DNA sequencing (Illumina Miseq Paired-End) and used a wide range of geochemical instruments to determine porewater dissolved and solid-phase geochemical and stabile isotopic gradients. All microbiological and geochemical data were compared to temperature gradients, which were determined using temperature probes and temperature loggers.
Timing and spatial scale	Sediment cores from three different research cruises (1998, 2009, 2015) were compared. The 10 sediment cores analyzed are from the periphery of two different hydrothermal vent fields, which differ in that one vent field has vertical advection of hydrothermal fluids through sediments, whereas the other vent field does not, and is thus diffusion-dominated.
Data exclusions	We excluded several DNA samples, which had gene copy numbers that were less than 3 times higher than those of extraction negative controls. In addition, we analyzed all DNA sequenced data and removed sequences of typical human laboratory and laboratory reagent contaminants.
Reproducibility	All microbiological and geochemical analyses were replicated through the analysis of high-resolution vertical sedimentary gradients, which demonstrate gradual and systematic changes in microbiological and geochemical gradients in relation to sediment depth and sediment temperature. Quantfications of microbial community size were replicated across two different users and laboratories, as well as with different PCR primer combinations.
Randomization	Microbial communities were analyzed in relation to detailed geochemical and temperature data, with the aim of identifying dependencies between microbiological communities in relation to sediment geochemical conditions and temperature. All samples obtained from these sediment cores were analyzed.
Blinding	Blinding was performed due to the fact that microbiological analyses and the majority of geochemical analyses were performed by different persons who were not aware of the goals of this particular study.

Did the study involve field work? ☒ Yes ☐ No

Field work, collection and transport

Field conditions	All samples were collected by push cores from a manned submarine or using multi-corers and gravity corers that were lowered to the seafloor from a research vessel. The detailed conditions of the study sites (environmental history, temperature, temperature gradient, sedimentation rate, fluid flow, organic and inorganic geochemical gradients, 13C-isotopic gradients of dissolved and inorganic microbial metabolites) are described in the study, and in some cases refer to previously published data.
Location	All samples are from Guaymas Basin, which is located in the central Gulf of Mexico. The longitudes and latitudes of the 10 sites are shown in Table of our manuscript and too long to list here.

Access & import/export	All research cruises were in Mexican waters with official permission from the Mexican government. As part of the agreement with the Mexican government, 2-6 Mexican scientists participated in each research cruise and obtained samples.
Disturbance	The sediment coring methods used only minimally disturb seafloor habitats. Previous evidence of sampling was absent in later years, when other research cruises revisited the sites sampled.

Reporting for specific materials, systems and methods

We require information from authors about some types of materials, experimental systems and methods used in many studies. Here, indicate whether each material, system or method listed is relevant to your study. If you are not sure if a list item applies to your research, read the appropriate section before selecting a response.

Materials & experimental systems

n/a	Involved in the study	
☒	☐	Antibodies
☒	☐	Eukaryotic cell lines
☒	☐	Palaeontology and archaeology
☐	☒	Animals and other organisms
☒	☐	Human research participants
☒	☐	Clinical data
☒	☐	Dual use research of concern

Methods

n/a	Involved in the study	
☒	☐	ChIP-seq
☒	☐	Flow cytometry
☒	☐	MRI-based neuroimaging

Animals and other organisms

Policy information about studies involving animals; ARRIVE guidelines recommended for reporting animal research

Laboratory animals	This study did not involve laboratory animals.
Wild animals	This study did not involve wild animals.
Field-collected samples	All sediment and sediment porewater samples were recovered under sterile conditions and immediately after sampling frozen at -20C (geochemical samples) or -80C (microbiological samples).
Ethics oversight	No ethical approval or guidance was required. This study only involved the study of sediment microorganisms.

Note that full information on the approval of the study protocol must also be provided in the manuscript.
